# The Pink Colour of Adrenalin Solutions

**Published:** 1907-06-08

**Authors:** 


### Points in Treatment.

### THE PINK COLOUR OF ADRENALIN SOLUTIONS.

Adrenalin solution is often an emergency medicine. It is essential, therefore, that it should be at hand ready for use; there is seldom time to send to the chemist's for a fresh sample of it when it is required in such conditions, and yet the interval between the purchase of a stock of it and the moment when it may be urgently required may be a long one. The question at once arises, How long will adrenalin solution remain potent.

The answer is that unless precautions are taken in the making of the solution it will deteriorate comparatively soon. Every medical man is familiar with the pink colour that appears in the previously colourless solution when it has been kept a little while; the development of the pink colour occurs even in stoppered bottles; and if the solution is in a bottle which is not tightly corked the pink will pass on into a deep brown. If adrenalin be added to warm distilled water and then shaken well and exposed to the air, the development of the colours—first pink and then brown—can be watched. It is sometimes stated that a solution of adrenalin which has become pink is still efficacious in its action; but it seems certain that it has deteriorated, apparently from oxidation of more or less of the adrenalin. This deterioration does not occur if the bottle of solution is quite full and is well stoppered; but once the bottle has been opened, and some of the solution used, the rest is almost certain to deteriorate, even if the stopper is firmly replaced, unless appropriate measures are adopted in preparing the solution. The best method of keeping the solution is to make it up with a very small quantity of sulphurous acid; this acid is also used in preserving eserine sulphate solutions in eye work, and the amount required is so small that it does not contraindicate the use of the fluids in ophthalmic practice. Mr. Horace Finnemore, Pharmacist to Guy's Hospital, advises that the prescription should be as follows:—

| | | |
|---|---|---|
| Adrenalin ... ... ... | 0.10 | parts |
| Chlor-butyl alcohol ... ... | 0.50 | ,, |
| Sodium chloride ... ... ... | 0.90 | ,, |
| Diluted hydrochloric acid ... | 0.25 | ,, |
| Sulphurous acid ... ... ... | 0.25 | ,, |
| Distilled water, to make up to ... | 100.00 | ,, |

The distilled water should be sterilised by boiling, and then allowed to cool; when nearly cold, the chlor-butyl alcohol and the sodium chloride should be added; the former of these is only soluble in cold water to the extent of 1 part in 200, so that the above will be a saturated solution; if any of the chlor-butyl alcohol crystals remain undissolved they should be filtered off from the final solution; it is to ensure that as many as possible of the crystals shall be dissolved that they are added whilst the distilled water is still tepid. When it is quite cold the diluted hydrochloric acid, the sulphurous acid, and the adrenalin are added to 25 parts of the liquid, and when these are dissolved the rest of the liquid is added and made up with sterilised, but cold, distilled water to 100 parts. The solution seems to keep well. The object of putting in each of the ingredients is as follows:—

The adrenalin, in order to make a 1 in 1,000 solution of the active principle.

The chlor-butyl alcohol because of its local anæsthetic properties.

The sodium chloride in order to make the liquid isotonic with the blood; that is to say, equivalent to "normal saline" solution.

The hydrochloric acid to convert the adrenalin into the more stable adrenalin chloride.

The sulphurous acid to prevent disintegration of the adrenalin chloride and formation of the pink colour on keeping the solution in stock.

